# Clinical and economic burden of invasive pneumococcal disease in adults: a multicenter hospital-based study

**DOI:** 10.1186/1471-2334-13-202

**Published:** 2013-05-04

**Authors:** Joon Young Song, Jun Yong Choi, Jin Soo Lee, In-Gyu Bae, Young Keun Kim, Jang Wook Sohn, Yu Mi Jo, Won Suk Choi, Jacob Lee, Kyung Hwa Park, Woo Joo Kim, Hee Jin Cheong

**Affiliations:** 1 Division of Infectious Disease, Department of Internal Medicine, Korea University Guro Hospital, 148 Gurodong-ro, Guro-gu, Seoul 152-703, Republic of Korea; 2 Asian Pacific Influenza Institute (APII), Seoul, Republic of Korea; 3 Division of Infectious Diseases, Department of Internal Medicine, Yonsei University College of Medicine, Yonsei, Seoul, Republic of Korea; 4 Division of Infectious Diseases, Department of Internal Medicine, Inha University School of Medicine, Incheon, Republic of Korea; 5 Division of Infectious Diseases, Department of Internal Medicine, Gyeongsang National University School of Medicine, Gyeongsang, Republic of Korea; 6 Division of Infectious Diseases, Department of Internal Medicine, Yonsei University, Wonju College of Medicine, Wonju, Republic of Korea; 7 Division of Infectious Disease, Department of Internal Medicine, Korea University Anam Hospital, 73 Inchon-ro, Seongbuk-gu, Seoul 136-705, Republic of Korea; 8 Division of Infectious Diseases, Department of Internal Medicine, Konyang University College of Medicine, Daejeon, Republic of Korea; 9 Division of Infectious Disease, Department of Internal Medicine, Korea University Ansan Hospital, 123 Jeokgeum-ro, Danwon-gu, Ansan-si, Gyeonggi-do 425-707, Republic of Korea; 10 Division of Infectious Diseases, Department of Internal Medicine, Hallym University College of Medicine, Chuncheon, Republic of Korea; 11 Division of Infectious Diseases, Department of Internal Medicine, Chonnam National University Medical School, Gwangju, Republic of Korea

**Keywords:** Cost of illness, Pneumococcal infection, Pneumococcal vaccines, *Streptococcus pneumoniae*

## Abstract

**Background:**

*Streptococcus pneumoniae* causes a broad spectrum of illnesses ranging from mild upper respiratory tract infections to invasive pneumococcal disease (IPD). Quantitative data on the burden of pneumococcal disease, important for the establishment of appropriate vaccination strategies, is currently lacking in adults.

**Methods:**

This multicenter, retrospective cohort study was designed to estimate the clinical and economic burden of IPD in adults over the last decade. Data were collected from patients with IPD at 10 university hospitals in South Korea. We estimated the proportion of IPD among all hospitalized patients, the case fatality rate, and the direct medical costs of IPD. Data were further analyzed according to age and risk groups.

**Results:**

During the study period, 970 patients with IPD were identified. The mean age for all patients was 60.9 years; patients aged 50–64 years (33.0%) were most numerous, followed by those aged 65–74 years (27.4%). Overall, the proportion of IPD was 0.36 cases/1000 hospitalized patients and the case fatality rate was 30.9%, which increased significantly with age (*p* < 0.01). The mean direct medical costs were estimated to be US $7,452 without a difference between age and risk groups. On multivariate analysis, old age, advanced ECOG performance status, bacteremic pneumonia, and nosocomial infection were independent risk factors of 30-day case fatality.

**Conclusions:**

The clinical disease burden of IPD increased significantly with age and direct medical costs from IPD were substantial, regardless of age and co-morbid conditions. The current age-based vaccination strategy appears to be appropriate.

## Background

*Streptococcus pneumoniae* causes a broad spectrum of illnesses ranging from mild upper respiratory tract infection to severe invasive disease. Of all the manifestations, invasive pneumococcal disease (IPD) is associated with a particularly high case fatality rate and economic cost. Although the incidence of IPDs has decreased in recent years with the introduction of the 7-valent pneumococcal conjugate vaccine (PCV7), the WHO has estimated that 1.6 million deaths are caused by pneumococci annually [1]; this estimate included the deaths of 0.7–1 million children aged less than 5 years, accounting for about 11% of all deaths in children aged 1–59 months [2]. In the United States, IPD was responsible for 22,000 deaths, 4 million disease episodes, and direct medical care costs of $3.5 billion in 2004 [3]. In this study, most of the deaths and medical costs were found to be associated with adult patients despite their accounting for only half of the disease episodes [3]. In adults, data on disease burden need to be further investigated with respect to age, underlying comorbidities, and performance status. These data could be useful in revising vaccination strategies.

Currently, both the 23-valent pneumococcal polysaccharide vaccine (PPV23) and PCV13 have been used for vaccination of adults. PPV23 is recommended for all adults aged ≥65 years and for adults at high risk who are aged 19–64 years [4]. A one-time revaccination dose of PPV23 is recommended 5 years after the first dose for asplenic or immunocompromised persons. In addition to PPV23, PCV13 was licensed for the prevention of IPD and pneumonia in adults aged ≥50 years [4]. PCV13 has been licensed for use in adults solely on the basis of immunogenicity data, and a clinical trial is underway to evaluate the efficacy of PCV13 against pneumococcal pneumonia in adults.

Despite the wide availability of pneumococcal vaccines, vaccination rates around the world remain suboptimal in adults (less than 50% even in high-risk groups) [5-7]. Among South Korean children, the PCV7 coverage rate increased up to 72% in 2009 [8], while the uptake rate in high-risk adults was very low (0.6%), according to a nationwide survey in South Korea [9]. Insufficient data on the burden of pneumococcal disease might be a barrier to increased immunization rates among the South Korean adults.

This study was designed to estimate the proportion of IPD among all hospitalized patients, the case fatality rate, and the direct medical costs associated with IPD in South Korean adults over the last decade. In order to better clarify IPD disease burden in adults, data were analyzed by age and risk group.

## Methods

### Study design and subjects

This retrospective cohort study included all patients with IPD at 10 university hospitals in South Korea between January 1, 2001 and September 30, 2011. To obtain data representative of the entire nation, participating hospitals were selected on the basis of geographic location and population served. We included hospitals only if all data were available regarding direct medical costs. All IPD cases among Korean adults aged ≥18 years were included. Available data were stratified according to age and risk group and analyzed to determine the proportion of IPD (cases/1000 hospitalized patients), distribution of primary infection sites, case fatality rate, length of hospital stay, and direct medical costs. Total annual hospital admissions were obtained from an electronic database system. We also aimed to evaluate the change in the proportion of patients with IPD and the case fatality rate after introduction of the PCV7 vaccine during the study period.

All cases that met the IPD definition were selected from microbiological databases. The patients’ medical records were reviewed to determine age, sex, comorbidities, Eastern Cooperative Oncology Group (ECOG) performance status, date of admission, primary site of infection, intensive care unit (ICU) admission, duration of hospitalization, and 30-day case fatality. The case fatality rate was estimated among patients whose survival or death was recorded. Direct medical costs were captured from information on resource use contained in the medical records (consultations, prescriptions, and procedures). This research was performed with approval from the institutional review boards of the selected 10 university hospitals. Written consent was waived by the institutional review board, because of the retrospective nature of the study and because patients' private information was not included.

### Definitions

IPD was defined as the isolation of *S*. *pneumoniae* from a normally sterile site, such as blood, cerebrospinal fluid (CSF), and pleural or ascites fluid. Each patient was classified into one of three groups based on underlying medical conditions: high-risk (those advised to receive PPV23 vaccination every five years), moderate-risk (those requiring a single dose vaccination in their life), and low-risk (those who are not recommended to receive pneumococcal vaccination). High risk was defined by the presence of one or more of the following: (1) splenic dysfunction including post-splenectomy status, (2) hematologic malignancy such as multiple myeloma, leukemia, or lymphoma, (3) a condition affecting the bone marrow or lymphatic system, such as chemotherapy with alkylating drugs or antimetabolites, or radiation within the previous 3 months, (4) solid organ or stem cell transplantation, (5) chronic renal disease such as nephrotic syndrome or chronic renal failure, (6) HIV infection, (7) high-dose corticosteroid use (≥20 mg/day of prednisone or equivalent) lasting two or more weeks, or (8) treatment with a recombinant human immunomodulator, such as rituximab, adalimumab, or infliximab. Moderate risk was defined by one or more of the following: (1) diabetes mellitus, (2) chronic liver disease, (3) chronic pulmonary disease, such as asthma or chronic obstructive lung disease, (4) chronic cardiovascular disease, such as heart failure, cardiomyopathy, or other chronic condition affecting cardiac function, (5) current smoking, or (6) heavy alcohol use, which was defined as alcohol use for more than 5 days a week or having a diagnosis of alcoholism. Low risk was assigned to conditions that did not satisfy criteria for high or moderate risk. IPD was defined as a nosocomial infection if the first positive culture was obtained more than 48 hours after hospital admission or if the patient had been hospitalized for more than 2 of 7 days before the first positive culture. Recurrent infection in a patient was defined as two or more IPD episodes within a 12-month period.

### Calculation of direct medical costs for invasive pneumococcal disease

The direct medical costs of IPD were extracted from the hospital financial database, which included both outpatient and inpatient costs: hospitalization expenditures, nursing, IPD-related prescription drugs, examination fees, etc. These were inflated to reflect 2011 prices. To capture direct medical costs only, we used the following approach. To estimate hospitalization costs, we excluded from the calculation (1) nosocomial IPD cases that occurred during hospitalization at the 10 study hospitals, and (2) costs from day 1 of the onset of comorbidity to the last day of hospitalization (if any comorbidity developed during the entire hospitalization period). Infectious disease specialists in each hospital reviewed all the medical records to find new comorbidities that required surgical/medical treatment unrelated to IPD. With respect to outpatient visits, we included outpatient costs for medications and procedures that were IPD-related. The estimated costs of disease were calculated in Korean won (KRW) and converted into US dollars at a rate of KRW1200=US$1.

### Antibiotic susceptibility test

Antibiotic susceptibility was tested in accordance with Clinical Laboratory Standards (CLSI) guidelines at each hospital [10]. Cases with available minimal inhibitory concentration (MIC) results were included in the analysis of antibiotic susceptibility. Interpretive breakpoints for penicillin (non-meningitis *S*. *pneumoniae* isolates), ≤2 μg/mL (susceptible), 4 μg/mL (intermediate), and ≥8 μg/mL (resistant); and for erythromycin, ≤0.25 μg/mL (susceptible), 0.25<MIC≤1 μg/mL (intermediate), and > 1 μg/mL (resistant).

### Statistical analysis

We performed descriptive analyses and comparisons to examine demographics, clinical characteristics, and IPD disease burden based on age and risk group. Data were expressed as the mean ± standard deviation and were analyzed using SPSS version 10.0 (SPSS Inc., Chicago, IL, USA). Chi-square tests were carried out to compare categorical variables. For continuous variables, either student‘s *t*-test or one-way analysis of variance (ANOVA) was performed with Tukey’s method for multiple comparisons. A *p*-value < 0.05 was considered statistically significant. Using a logistic regression model, multivariate analysis was carried out to identify independent risk factors for 30-day case fatality.

## Results

### Demographic and clinical characteristics of invasive pneumococcal disease

Nine hundred seventy patients with IPD were identified during the study period, including 264 (27.2%) at high risk, 423 (43.6%) at moderate risk, and 283 (29.2%) at low risk. The mean age of patients was 60.9 years. Patients aged 50–64 years were most numerous (33.0%), followed by those aged 65–74 years (27.4%), 18–49 years (22.2%), and 75 years and older (17.4%). The demographic and clinical characteristics of patients with IPD are presented in Table 1. The age groups differed significantly in gender ratio, alcohol use, smoking, ECOG performance status, risk group distribution, and underlying conditions. Male sex, heavy alcohol use, and current smoking were common in the group aged 18–49 and in the group aged 50–64 years, while the proportion with ECOG performance status grade 4 increased with age. Patients at high and moderate risk were most numerous in the group aged 50–64 years. Chronic pulmonary, cardiovascular, cerebrovascular, and neurodegenerative diseases, and conditions such as multiple myeloma increased in prevalence with age. The proportion of patients with diabetes mellitus increased significantly by age 50 (*p* < 0.01), while chronic liver disease was more common in the younger age groups (18–49 years and 50–64 years, *p* < 0.01). Patients receiving chemotherapy were predominantly in the two groups aged 50–64 and 65–74 years (*p* < 0.01).

**Table 1:** **Baseline patient characteristics and disease burden of invasive pneumococcal disease**, **stratified by age group**

	**Total (*n *= 970)**	**18-49 Years (*n *= 215)**	**50-64 Years (*n *= 320)**	**65-74 Years (*n *= 266)**	**≥75 Years (*n *= 169)**	***p*-value**
Age, mean±SD	60.9±15.0	38.8±7.9	58.2±4.1	69.3±2.7	81.0±4.4	< 0.01
Risk group, no. (%)						< 0.01
High risk	264 (27.2)	38 (17.7)	112 (35.0)	86 (32.3)	28 (16.6)	
Moderate risk	423 (43.6)	91 (42.3)	147 (45.9)	108 (40.6)	77 (45.5)	
Low risk	283 (29.2)	86 (40.0)	61 (19.1)	72 (27.1)	64 (37.9)	
Sex (male), no. (%)	673 (69.4)	139 (64.7)^a^	237 (74.1)^b^	191 (71.8)^b^	106 (62.7)^a^	0.02
ECOG performance status grade 4, no. (%)	89 (9.2)	10 (4.7)^a^	19 (5.9)^a^	27 (10.2)^b^	33 (19.5)^c^	< 0.01
Heavy alcoholic, no. (%)*	99 (10.2)	32 (14.9)^a^	46 (14.4)^a^	14 (5.3)^b^	7 (4.1)^b^	< 0.01
Current smoker, no. (%)	171 (17.6)	46 (21.4)^a^	60 (18.8)^a^	48 (18.0)^a^	17 (10.1)^b^	0.03
Comorbid condition, no. (%)	630 (64.9)	95 (44.2)^a^	235 (73.4)^b^	188 (70.7)^b^	112 (66.3)^b^	< 0.01
Splenic dysfunction	8 (0.8)	4 (1.9)	1 (0.3)	3 (1.1)	0 (0)	0.14
Multiple myeloma	38 (3.9)	2 (0.9)^a^	23 (7.2)^b^	10 (3.8)^b^	3 (1.8)^ab^	< 0.01
Other hematologic malignancy	26 (2.7)	7 (3.3)^a^	8 (2.5)^b^	8 (3.0)^b^	3 (1.8)^a^	0.81
Chemotherapy	163 (16.8)	18 (8.4)	74 (23.1)	55 (20.7)	16 (9.5)	< 0.01
Solid organ/stem cell transplantation	6 (0.6)	3 (1.4)	2 (0.6)	0 (0)	1 (0.6)	0.29
Long-term steroid therapy	11 (1.1)	4 (1.9)	3 (0.9)	2 (0.8)	2 (1.2)	0.69
Chronic renal failure	54 (5.6)	9 (4.2)	20 (6.3)	17 (6.4)	8 (4.7)	0.65
Nephrotic syndrome	2 (0.2)	1 (0.5)	1 (0.3)	0 (0)	0 (0)	0.62
HIV infection	1 (0.1)	0 (0)	0 (0)	1 (0.4)	0 (0)	0.45
Diabetes mellitus	206 (21.2)	23 (10.7)^a^	78 (24.4)^b^	60 (22.6)^b^	45 (26.6)^b^	< 0.01
Chronic liver disease	133 (13.7)	31 (14.4)^a^	71 (22.2)^b^	23 (8.6)^c^	8 (4.7)^c^	< 0.01
Chronic pulmonary disease	81 (8.4)	4 (1.9)^a^	23 (7.2)^b^	25 (9.4)^b^	29 (17.2)^c^	< 0.01
Chronic cardiovascular disease	76 (7.8)	9 (4.2)^a^	17 (5.3)^a^	22 (8.3)^a^	28 (16.6)^b^	< 0.01
Cerebrovascular disease	58 (6.0)	9 (4.2)^a^	12 (3.8)^a^	23 (8.6)^b^	14 (8.3)^ab^	0.03
Neurodegenerative disease	14 (1.4)	0 (0)^a^	2 (0.6)^a^	5 (1.9)^ab^	7 (4.1)^b^	< 0.01
Neuromuscular disease	9 (0.9)	2 (0.9)	2 (0.6)	2 (0.8)	3 (1.8)	0.37
Clinical presentation, no. (%)						
Primary bacteremia	190 (19.6)	40 (18.6)^ab^	75 (23.4)^a^	55 (20.7)^a^	20 (11.8)^b^	0.02
Bacteremic pneumonia	538 (55.5)	102 (47.4)^a^	160 (50.0)^a^	150 (56.4)^a^	126 (74.6)^b^	< 0.01
Empyema	49 (5.1)	4 (1.9)^a^	14 (4.4)^a^	25 (9.4)^b^	6 (3.6)^a^	< 0.01
Meningitis	84 (8.7)	35 (16.3)^a^	21 (6.6)^b^	20 (7.5)^b^	8 (4.7)^b^	< 0.01
Peritonitis	74 (7.6)	23 (10.7)^a^	33 (10.3)^a^	13 (4.9)^b^	5 (3.0)^b^	< 0.01
Septic arthritis	13 (1.3)	1 (0.5)	5 (1.6)	3 (1.1)	4 (2.4)	0.42
Infective endocarditis	2 (0.2)	1 (0.5)	1 (0.3)	0 (0)	0 (0)	0.62
Time from symptom onset to admission (days), mean±SD	3.5±8.5	4.0±9.0	2.8±4.7	4.1±12.5	2.9±4.6	0.22
Recurrent infection, no. (%)	8 (0.8)	2 (0.9)	5 (1.6)	1 (0.4)	0	0.24
Site of acquisition of infection, no. (%)						< 0.01
Community	794 (81.9)	191 (88.8)	265 (82.8)	215 (80.8)	123 (72.8)	
Long-term care facility	42 (4.3)	4 (1.9)	7 (2.2)	7 (2.6)	24 (14.2)	
Tertiary referral hospital	134 (13.8)	20 (9.3)	48 (15.0)	44 (16.6)	22 (13.0)	
Proportion of IPD (cases/1,000 hospitalized patients)	0.36	0.21^a^	0.39^b^	0.49^c^	0.69^d^	< 0.01
Case fatality rate, no. (%)^†^	281/909 (30.9)	38/207 (18.4)^a^	83/309 (26.9)^a^	86/243 (35.4)^b^	74/150 (49.3)^c^	< 0.01
Direct medical costs per case‡						0.11
Mean (95% CI)	US $7,452	US $6,223	US $8,191	US $7,910	US $6,956	
	(6,816-8,088)	(5,218-7,229)	(6,963-9420)	(6,645-9,175)	(5,351-8,561)	
Median (IQR)	US $4,772	US $4,092	US $5,095	US $5,320	US $4,439	
	(2,378-8,698)	(2,364-7,844)	(2,898-9,018)	(2,796-10,590)	(1,844-8,136)	

ECOG, Eastern Cooperative Oncology Group; IQR, inter-quartile range.

NOTE: The same letters (alphabetical superscripts) indicate non-significant differences between groups based on either Tukey’s multiple comparison tests or chi-square tests.

* Defined as alcohol use for more than 5 days a week or having a diagnosis of alcoholism.

^†^Case fatality rates were based only on patients whose survival or death was confirmed.

^‡^KRW1200=US$1.

In assessing clinical presentation according to age, bacteremic pneumonia and empyema were more common in adults 65 years of age and older than in younger adults, while meningitis and peritonitis were more common in the younger groups (*p* < 0.01) (Table 1). Patients were hospitalized after a mean of 3.5 days from symptom onset. Recurrent infection was rare (0.8%) irrespective of age group.

The proportion of IPD acquired at either a long-term care facility or tertiary referral hospital increased significantly with increasing patient age (*p* < 0.01). Overall, 18.1% of the infections were nosocomial, increasing to 19.2% in subjects aged 65–74 years, and to 27.2% in subjects aged 75 years and older.

### Disease burden of invasive pneumococcal disease

Overall, the proportion of hospitalized patients with IPD was 0.36 cases/1000 hospitalized, and the case fatality rate was 30.9%, which increased significantly with age (*p* < 0.01) (Table 1). However, the direct medical costs per case of IPD did not differ between age groups (mean cost US $7,452/case; *p* = 0.11) (Table 1).

Table 2 shows the clinical and economic burden associated with invasive pneumococcal disease. Stratification according to risk group revealed no significant differences in the duration of hospitalization (22.0 days vs. 17.8 days vs. 19.0 days, *p* = 0.47), case fatality rate (35.3% vs. 27.7% vs. 31.5%, *p* = 0.28), and direct medical cost per case (mean, US$ 7,667 vs. US$ 7,037 vs. US$ 7,903; *p* = 0.58) among the three risk groups (high risk vs. moderate risk vs. low risk). However, both the rate of admission and the duration of stay in the intensive care unit (ICU) were higher in the low- and moderate-risk groups compared to the high-risk group (*p* < 0.01 and *p* = 0.05, respectively) reflecting aggressive therapy in previously healthy subjects. On further analysis by age in each risk group, the case fatality rate tended to significantly increase with age in the high- and low-risk groups (*p* < 0.01). Direct medical costs differed by age only in the moderate-risk group; costs were higher in patients aged 50–64 years compared to the others (*p* = 0.03) (Table 2).

**Table 2:** **Burden of invasive pneumococcal disease**, **stratified by risk group**

	**High-risk group**					***p*-value**
	**Total (*n *=264)**	**18-49 Years (*n *=38)**	**50-64 Years (*n *=112)**	**65-74 Years (*n *=86)**	**≥75 Years (*n *=28)**	
Duration of hospitalization	20.1±19.3	22.0±16.5	21.7±21.9	17.3±14.4	19.4±24.2	0.41
ICU admission, no. (%)	84 (31.8)	13 (34.2)	33 (29.5)	28 (32.6)	10 (35.7)	0.9
Duration of ICU admission	3.4±8.7	3.2±7.2	2.7±6.2	3.1±7.5	6.7±17.8	0.19
Case fatality rate, no. (%)*	89/252 (35.3)	8/38 (21.1)^a^	33/107 (30.8)^a^	30/82 (40.0)^a^	18/25 (72.0)^b^	< 0.01
Time to death (days)	9.7±8.5	9.6±5.9	10.6±9.1	8.6±7.9	9.7±9.9	0.85
Direct medical costs per case†						0.76
Mean (95% CI)	US $7,667	US $6,990	US $7,135	US $8,609	US $7,880	
	(6,395-8,938)	(4,464-9,516)	(5,746-8,524)	(5,648-11,569)	(2,297-13,462)	
Median (IQR)	US $5,552	US $6,749	US $5,612	US $5,472	US $3,262	
	(2,567-9,872)	(2,474-9,948)	(3,007-9,650)	(3,278-10,442)	(1,532-10,034)	
	**Moderate-risk group**					***p*-value**
	**Total (*n *=423)**	**18-49 years (*n *=91)**	**50-64 years (*n *=147)**	**65-74 years (*n *=108)**	**≥75 years (*n *=77)**	
Duration of hospitalization	17.8±26.1	15.3±15.7	17.7±17.3	19.0±24.4	19.0±45.3	0.76
ICU admission, no. (%)	191 (45.2)	35 (38.5)	68 (46.3)	46 (42.6)	42 (54.5)	0.19
Duration of ICU admission	5.3±20.8	3.2±6.4	5.2±10.9	4.7±8.9	9.0±44.9	0.33
Case fatality rate, no. (%)*	110/397 (27.7)	19/88 (21.6)	37/144 (25.7)	28/96 (29.2)	26/69 (37.7)	0.14
Time to death (days)	7.4±6.8	6.3±5.3	8.8±7.7	7.1±6.1	6.5±6.9	0.45
Direct medical costs per case†						0.03
Mean (95% CI)	US $7,037	US $5,404^a^	US $8,756^b^	US $6,964^ab^	US $5,730^a^	
	(6,120-7,955)	(4,191-6,617)	(6,650-10,861)	(5,395-8,532)	(4,423-7,037)	
Median (IQR)	US $4,517	US $3,685	US $4,956	US $4,664	US $4,679	
	(2,432-7,961)	(2,405-6,805)	(2,797-10,003)	(2,402-9,499)	(1,879-7,057)	
	**Low-risk group**					***p*-value**
	**Total (*n *=283)**	**18-49 years (*n *=86)**	**50-64 years (*n *=61)**	**65-74 years (*n *=72)**	**≥75 years (*n *=64)**	
Duration of hospitalization	19.0±25.6	19.0±20.9	21.9±21.2	19.9±27.4	15.4±32.3	0.55
ICU admission, no. (%)	139 (49.1)	33 (38.4)	31 (50.8)	40 (55.6)	35 (54.7)	0.11
Duration of ICU admission	6.8±13.8	5.9±13.6	8.3±14.3	6.8±13.1	6.8±14.7	0.78
Case fatality rate, no. (%)^*^	82/260 (31.5)	11/81 (13.6)^a^	13/58 (22.4)^a^	28/65 (43.1)^b^	30/56 (53.6)^b^	< 0.01
Time to death (days)	7.9±9.0	10.2±15.4	9.2±10.3	9.3±8.1	5.3±5.2	0.26
Direct medical costs per case†						0.69
Mean (95% CI)	US $7,903	US $6,849	US $8,528	US $8,557	US $8,090	
	(6,675-9,131)	(4,931-8,766)	(5,715-11,342)	(6,239-10,875)	(4,727-11,453)	
Median (IQR)	US $4,968	US $4,448	US $5,726	US $5,896	US $3,367	
	(2,187-9,613)	(1,856-8,574)	(3,443-8,858)	(2,178-11,733)	(1,879-9,308)	

IQR, inter-quartile range.

NOTE: The same letters (alphabetical superscripts) indicate non-significant differences between groups based on either Tukey’s multiple comparison tests or chi-square tests.

^*^ Case fatality rates were based only on patients whose survival or death was confirmed.

†KRW1200=US$1.

### Prognostic factors of invasive pneumococcal disease

On univariate analysis, older age, advanced ECOG performance status, bacteremic pneumonia, and nosocomial infection were associated with a higher 30-day case fatality rate (*p* < 0.05). Risk group (*p* = 0.12), sex (*p* = 0.08), and antibiotic susceptibility (penicillin, *p* = 0.28; erythromycin, p = 0.73) did not influence the 30-day case fatality rate. Meningitis was associated with a lower case fatality rate (*p* < 0.01). On multivariate analysis, old age (odds ratio, 1.02; 95% CI 1.01-1.03), ECOG performance status grade 4 (odds ratio, 5.97; 95% CI 3.30-10.80), bacteremic pneumonia (odds ratio, 1.53; 95% CI 1.10-2.14), and nosocomial infection (odds ratio, 2.21; 95% CI 1.49-3.25) were independent risk factors for 30-day case fatality (Table 3).

**Table 3:** **Prognostic factors related to 30**-**day mortality among patients with invasive pneumococcal disease**

**Variables**	**0dds ratio**	**95% Confidence interval**
Age	1.02	1.01-1.03
Sex (male)	1.17	0.83-1.65
ECOG performance status: reference category, grade 0		
Grade 1	1.09	0.73-1.63
Grade 2	1.1	0.68-1.78
Grade 3	1.42	0.85-2.39
Grade 4	5.97	3.30-10.80
Risk group: reference category, low-risk group		
Moderate risk	0.87	0.81-1.87
High risk	1.23	0.59-1.27
Bacteremic pneumonia	1.53	1.10-2.14
Meningitis	0.57	0.27-1.21
Nosocomial infection	2.21	1.49-3.25

ECOG, Eastern Cooperative Oncology Group.

### Trends in proportion, case fatality rate, and antibiotic susceptibility

**Figure 1:**
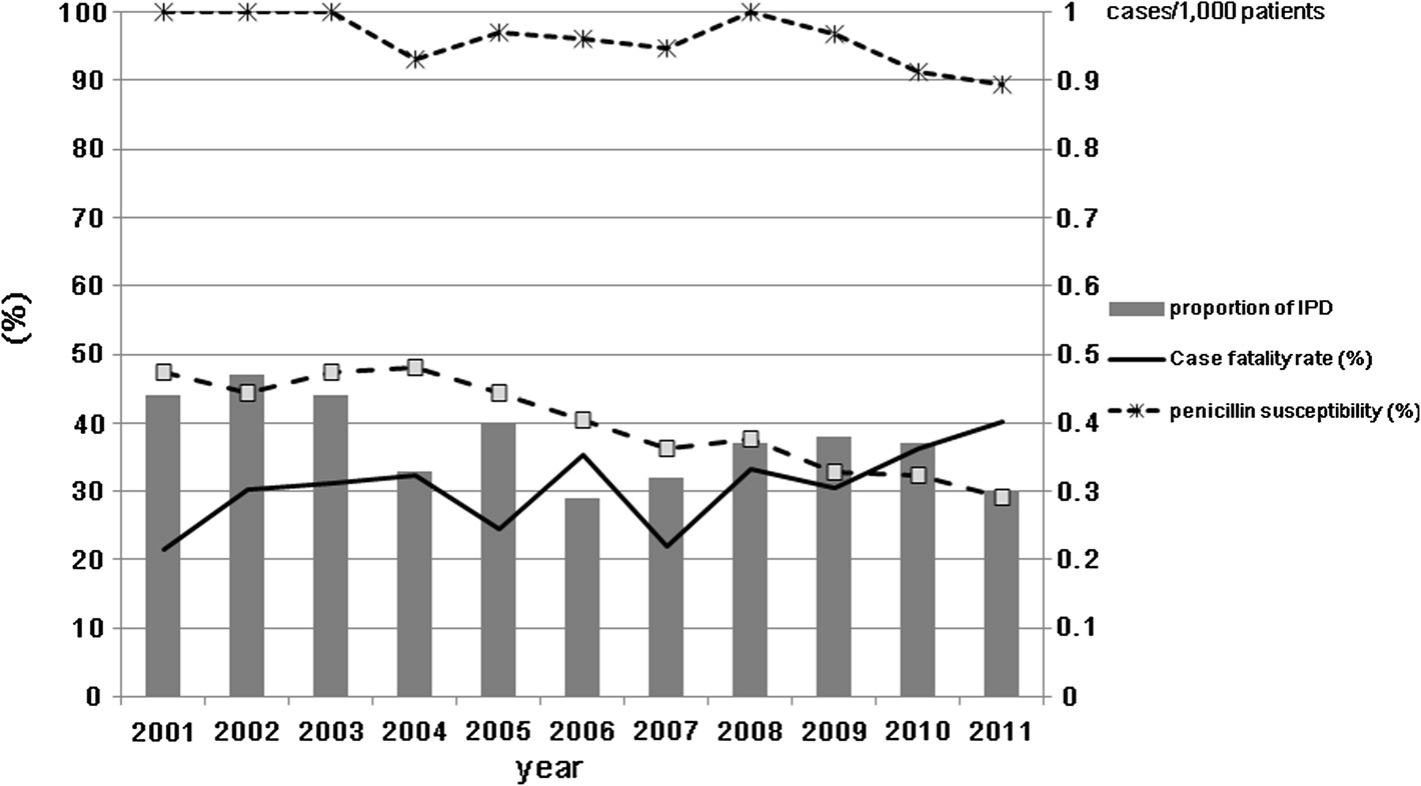
**Trends in invasive pneumococcal disease (IPD): proportion of IPD,case fatality rate**, **and antibiotic susceptibility.**

Over the study period, the proportion of IPD among hospitalized patients (cases/1000), the case fatality rate, and the antibiotic susceptibility rate were serially estimated (Figure 1). Since the introduction of PCV7 in 2003, the proportion of IPD and case fatality rates did not change remarkably. However, rates of penicillin and erythromycin susceptibility decreased in the years leading up to and after 2010: 100% (2008), 96.8% (2009), 91.3% (2010) and 89.5% (2011) for penicillin; 37.7% (2008), 32.7% (2009), 32.4% (2010) and 29.1% (2011) for erythromycin.

## Discussion

Although the global burden of IPD is described extensively in the literature, most studies focus on pediatric diseases, and currently available data for Asian countries is lacking. This multicenter study evaluated the IPD disease burden based on laboratory-confirmed cases in adults, stratified by age and underlying conditions. Direct medical costs were estimated by an objective, concrete method to avoid overestimation. These results may be useful for evaluating the cost-effectiveness of pneumococcal vaccines in the future.

Consistent with previous studies [11-13], the proportion of IPD among hospitalized patients and the IPD case fatality rate increased with age. Though patients aged 65 years and older may be more likely to develop IPD compared to younger patients, those aged 50–64 years comprised the largest percentage in this study. A majority of patients aged 50–64 years (73.4%, 239 among 320 subjects) had comorbidities such as diabetes and chronic lung or cardiovascular diseases. Weycker et al.[13] previously found that persons aged 50–64 years comprised the largest proportion of the IPD patients. Considering that this age group is most active socially, indirect medical costs may be greater than corresponding costs for the elderly. In addition, Weycker et al. [13] reported that the majority of pneumococcal diseases (≥60%), pneumococcal-related death, and pneumococcal-related costs were accounted for by high-risk cases. The high-risk group may be also at greatest risk for IPD. However, contrary to the study by Weycker et al. [13], the moderate-risk group in our study accounted for more than 40% of IPD cases. Pneumococcal vaccination for the group at moderate risk should be emphasized.

It is also noteworthy that even the low-risk group presented a substantial clinical and economic burden. This may be related to the presence of underlying cerebrovascular, neurocognitive, and neuromuscular disorders and poor performance status in this group of patients. Jain et al. reported that patients with neurologic diseases are at high risk for pneumonia after influenza infection [14]. Though currently not considered high priority for pneumococcal vaccination, patients with neurologic disease were at great risk for IPD. Moreover, several studies have shown that neurologic diseases are risk factors for mortality after pneumococcal infection [15,16]. This suggests that the definitions for low, moderate, and high risk groups should be reconsidered.

Similar to the findings of Weycker et al. [13], the direct medical costs per case did not differ significantly between the risk or age groups in the present study; however, our estimates (mean, US$ 5,404-8,756/case) were much lower than those reported by Weycker et al. (US$ 15,402-31,849/case). First, this difference may be related to the methods used for estimation. Weycker et al. [13] estimated the direct medical costs based on data from the 2004 Healthcare Cost and Utilization Project (HCUP) Nationwide Inpatient Sample (NIS) using mean age- and risk-specific charges, while we calculated them by adding only IPD-related costs for each patient. Second, direct medical costs may potentially be affected by the healthcare system and geographic factors that affect accessibility. In a US study by Huang et al. [3], mean direct costs per case of pneumococcal bacteremia was estimated to be US $12,667, which is also higher than our estimates.

The patients’ clinical presentations varied significantly by age and risk group. Primary bacteremia was common in the high-risk group, bacteremic pneumonia and peritonitis were most common in the moderate-risk group, and meningitis was prominent in the low-risk group. Bacteremic pneumonia or empyema was more common in older patients, while meningitis and peritonitis were more common in the younger adults. These differences may be related to host-specific serotype distributions, host immunity, or other factors. Previous reports showed that some serogroups with high IPD potential (1, 5 and 7) affected relatively healthy adults, while those with low or intermediate IPD potential (3, 6, 8, 15, 19, 23, 33 and 38) were more likely to affect individuals with immunocompromise due to age and/or comorbidity [17,18]. Serotypes 3 and 19A pneumococci are more likely to cause pneumonia/empyema [19], while Serotype 1 is known to cause pneumonia and peritonitis in young adults [20].

In this study, the overall case fatality rate (30.9%) was higher than in previous reports, which may reflect that the study was composed exclusively of adults. Similarly in a previous Korean study, the case fatality rate for bacteremic pneumonia was 28.6% in adults [16]. In contrast, the case fatality rates for children were reported to be lower than 15% [21]. Independent risk factors for 30-day case fatality include advanced age, poor performance status, bacteremic pneumonia, and nosocomial infection. The case fatality in this study was not related to primary bacteremia, but to bacteremic pneumonia. Previous studies reported advanced age, male sex, comorbidities, alcoholism, sepsis, and meningitis as risk factors for mortality [17,19]. In this study, meningitis was not related to survival, contrary to previous studies. This may be related to the age of the study population. The risk of meningitis is much higher in pediatric patients than in adults, and infants under one year of age have an especially high case fatality rate [19].

Since the introduction of the PCV7 vaccine in 2003, a substantial decline in IPD incidence has been reported in the target population aged 5 years and younger [22]. In a study conducted in the Netherlands, however, the overall IPD incidence remained constant in adults [23]. With a serotype shift under the vaccine pressure, the PCV7 coverage rate fell below 25% in Korean patients with pneumococcal pneumonia irrespective of age (unpublished data). Likewise, the proportion of hospitalized adult patients with IPD and the adult case fatality rate did not decrease remarkably over this decade according to this study. Interestingly, the rates of bacterial susceptibility to penicillin and erythromycin decreased around 2010, which may be related to the expansion of the multidrug-resistant serotype 19A [11,24,25].

This study has several limitations. First, we could not estimate the incidence of IPD because the study populations were highly mobile and IPD patients were referred from diverse areas of Korea. Second, the serotype distribution could not be investigated. Third, this study was conducted retrospectively, introducing a potential information bias. Uncontrolled confounding factors might exist. To minimize these factors, well-trained research nurses collected data in each hospital using a structured case report form. All data were reviewed and processed without patient-identifying information at the data management center under the supervision of a medical advisor.

## Conclusion

In conclusion, we investigated the clinical and economic burden of IPD in adults. The proportion of IPD among hospitalized patients and the case fatality rate increased significantly with age, and direct medical costs were high regardless of age and comorbid conditions. The current age-based vaccination strategy appears appropriate, however, given the results of these studies. Patients aged 50–64 years comprised the greatest part of the IPD population, and most of these patients had comorbid conditions, placing them at higher risk. Further studies are indicated to evaluate the cost-effectiveness of pneumococcal vaccination targeting adults aged 50 years and older.

## Competing interest

The authors have no conflicts of interest to disclose.

## Authors’ contributions

JYS and HJC participated in study design, analyzed the data and drafted the manuscript. JYC, JSL, In-GB, YKK, JWS, YMJ, WSC, JL, KHP and WJK collected and analyzed the data. All authors read and approved the final manuscript.

## Pre-publication history

The pre-publication history for this paper can be accessed here:

http://www.biomedcentral.com/1471-2334/13/202/prepub
